# Acute Kidney Disease following Acute Kidney Injury in Children—A Retrospective Observational Cohort Study on Risk Factors and Outcomes

**DOI:** 10.3390/jcm13113145

**Published:** 2024-05-27

**Authors:** Flavia Chisavu, Lazar Chisavu, Viviana Ivan, Adalbert Schiller, Adelina Mihaescu, Luciana Marc, Ramona Stroescu, Ruxandra Maria Steflea, Mihai Gafencu

**Affiliations:** 1 Department of Paediatric Nephrology, “Louis Turcanu” Emergency County Hospital for Children, Rue Iosif Nemoianu, Number 2, 300041 Timisoara, Romania; farkas.flavia@umft.ro (F.C.); stroescu.ramona@umft.ro (R.S.); steflea.ruxandra@umft.ro (R.M.S.); mgafencu@umft.ro (M.G.); 2 Centre for Molecular Research in Nephrology and Vascular Disease, Faculty of Medicine “Victor Babes”, 300041 Timisoara, Romania; chisavu.lazar@umft.ro (L.C.); schiller.adalbert@umft.ro (A.S.); mihaescu.adelina@umft.ro (A.M.); marc.luciana@umft.ro (L.M.); 3 Discipline of Nephrology from University of Medicine and Pharmacy “Victor Babes”, 300041 Timisoara, Romania; 4 Discipline of Cardiology from University of Medicine and Pharmacy “Victor Babes”, 300041 Timisoara, Romania; 5 Discipline of Paediatrics from University of Medicine and Pharmacy “Victor Babes”, 300041 Timisoara, Romania

**Keywords:** acute kidney disease, children, mortality, chronic kidney disease

## Abstract

**Background**: Acute kidney disease (AKD) is a known risk factor for increased mortality and evolution towards chronic kidney disease (CKD) in adults. The data regarding AKD in children are scarce. The purpose of our study was to explore the risk factors for developing AKD based on exposures and susceptibilities in children with AKI doubled by the biological parameters from the first day of identified AKI. In addition, we followed the trajectory of AKD following an acute kidney injury (AKI) episode in children during hospital admission and after discharge with special considerations towards mortality and progression to new-onset CKD. **Methods**: We retrospectively evaluated 736 children, ages between 2 and 18 years old, with identified AKI during hospital admission in a tertiary care hospital from west Romania over a 9-year period. **Results**: AKD incidence following an AKI episode was 17%. Patients who developed AKD were older, with higher baseline serum creatinine, urea, C reactive protein and lower proteins, haemoglobin and sodium levels. In the adjusted model, no biological parameters influenced AKD development. Regarding certain exposures and personal susceptibilities in children with AKI, only anaemia independently increased the risk of AKD development by 2.47 times. However, out of the AKI causes, only the intrinsic causes of AKI independently increased the risk of progressing to AKD (glomerulonephritis by 4.94 and acute tubule-interstitial nephritis by 2.76 times). AKD increased the overall mortality by 2.6 times. The factors that independently increased the risk of CKD were AKD, acute tubular necrosis and higher baseline serum creatinine values. **Conclusions**: Only anaemia, glomerulonephritis and acute tubule-interstitial nephritis increased the risk of AKD development in children with AKI. AKD was an independent risk factor for mortality and new-onset CKD in children.

## 1. Introduction

Acute kidney injury (AKI) is an already proven risk factor for worse kidney outcomes in adults and children with high mortality and increased hospital stay [1,2,3,4,5]. The evolution of AKI is multidirectional and most often linked to the underlying disease severity. AKI can evolve towards resolution of the episode during the first 7 days or it can progress towards acute kidney disease (AKD) or chronic kidney disease (CKD) [1,3]. The concept of AKD was first introduced by Kidney Disease Improving Global Outcomes (KDIGO) in the AKI guideline from 2012 [6]. The Acute Disease Quality Initiative (ADQI) 16 Workgroup further refined and staged AKD as a subacute or acute loss of kidney function with a duration between 7 and 90 days from the initial AKI event based on the KDIGO AKI criteria [7].

Since the concept of AKD was introduced in clinical studies as a prolonged AKI episode with high morbidity and mortality, it proved that the relationship between AKI, AKD and CKD represents a continuum of kidney dysfunction or time-dependent damage following an AKI episode. The AKI-AKD-CKD continuum has been studied in adult settings [8,9,10,11]. The latest meta-analysis in adults by Su showed that AKD is an independent risk factor for mortality, CKD development and kidney failure [12]. However, only a few studies evaluated the evolution of AKI towards AKD in children [13,14,15,16,17]. Three of the aforementioned studies evaluated AKD incidence and outcomes in specific groups of children, either in transplantation (allogenic hematopoietic stem cell) [16] or non-kidney solid organ transplantation [14] or following cardiopulmonary by-pass [15]. The latter two studies, by Deng [17] and Patel [13], evaluated AKD incidence and outcomes in a mixed group of paediatric patients aged between 1 month and 18 years old.

As kidney function evolution after birth is extremely dynamic, glomerular and tubular function immediately increases postnatally [18]. The dynamic changes in kidney function occur differently in children born preterm or with lower gestational ages. Nevertheless, children reach a glomerular filtration rate equivalent to adults around 2 years of age [19]. Given the high variability of kidney function in the first 2 years of life, we conducted a retrospective cohort study in children with stable and mature kidney function who developed AKD following an AKI episode. In addition, we evaluated the risk factors, outcomes and the risk of CKD development in children with AKD.

## 2. Material and Methods

### 2.1. Study Design

We conducted a retrospective observational cohort study in children between 2 and 18 years old, admitted in a tertiary care children’s hospital from west Romania. We analyzed and screened for all AKI admissions during 2014 and 2022. We included only the patients with AKI in the analysis of AKD and CKD. Inclusion criteria were at least two serum creatinine measurements during the first 7 days for the diagnosis of AKI, patients with AKI over 7 days who had at least one serum creatinine measurement during the first 7 days of AKD, and for new-onset CKD, a follow-up in serum creatinine 90 days after the initial AKI episode. Data were retrieved from the Electronically Data System of the hospital. Two independent doctors evaluated the data and AKI diagnosis was established in children who met the KDIGO serum creatinine criteria. Follow-up was performed until death or the last recorded admission. The estimated glomerular filtration rate (eGFR) was calculated using Bedside Schwartz formula for the lowest serum creatinine value [20]. The final cohort comprised a mixed paediatric population, from intensive and non-intensive care units. The Ethics Committee of the “Louis Turcanu” Children’s Hospital approved the study. Informed consent was waived due to the retrospective nature of the study. However, all legal guardians consented prior to hospital admission.

### 2.2. Outcomes and Definitions

The primary outcomes of the study were to determine AKD incidence following an AKI episode and to evaluate the risk factors associated with AKD development in children after reaching the adult kidney function. In addition, we evaluated the influence of AKD on in-hospital and post-discharge mortality, and the impact of AKD on new-onset CKD. The impact of AKD on mortality was evaluated only in patients who survived the first 7 days of AKI and reached the theoretical landmark of AKD development. The baseline serum creatinine was considered the lowest value prior to the AKI episode. If the patient did not have a previous serum creatinine value recorded, we considered baseline serum creatinine the lowest value of serum creatinine recorded after the AKI episode. AKI was defined using the KDIGO AKI criteria for serum creatinine [6]. If a patient presented multiple AKI episodes, we only included the first recorded AKI episode in our analysis. A later AKI episode was considered as subsequent AKI. Acute kidney disease was defined and staged based on the 2021 KDIGO recommendations using the serum creatinine value recorded after day 7 of AKI [21]. The diagnosis of new-onset CKD was based on the 2012 KDIGO guideline using the following criteria: persistence of kidney damage markers for more than 3 months (albuminuria, proteinuria, etc) or eGFR < 60 mL/min/1.73 sm for more than 3 months [22]. The impact of AKD on new-onset CKD development was performed only in patients with a follow-up longer than 3 months and after excluding the patients with pre-exiting CKD.

### 2.3. Variables of Interest

All the data were retrieved from the electronic data system of the hospital. We noted gender, environment, age, weight, height, length of stay and biological parameters measured in the first day of identified AKI. In addition, we retrieved data regarding intensive care unit (ICU) admission and the presence of the exposures and susceptibilities in patients with AKI. In addition, the exposures and susceptibilities were further analyzed in patients with AKD in order to obtain a better image of AKD outcomes in children.

### 2.4. Study Size

Out of 142,762-hospital admissions, 2765 episodes of AKI were identified. We first excluded the subsequent AKI episodes and the cohort comprised 2346 patients. We further excluded the patients between 1 day and 1.99 years old; thus, the final cohort comprised 736 patients. The analysis on mortality was performed on 720 patients, after excluding 16 children who died during the first 7 days of AKI. Evaluation of CKD development was performed in patients with more than 3 months follow-up, after excluding the pre-existent CKD, in 313 patients.

### 2.5. Statistical Analysis

The continuous variables were tested for normality using the Shapiro–Wilk test. Because none of our continuous variables followed a normal distribution, we defined median and interquartile range. The categorical variables were defined as numbers and percentages. For continuous variables, we used the Mann–Whitney test if there were two groups and Kruskal–Wallis for three or more comparison groups. The crude analysis was performed by calculating the odds ratio and 95% confidence interval. The adjusted analysis was performed using multivariable logistic regression model with a backwards selection of the variables (excluded if *p* > 0.1 and included for *p* < 0.05). The crude analysis for CKD development was performed using Kaplan–Meier survival curves. For the adjusted analysis on CKD development, we used a Cox proportional-hazards regression model. A *p* value less than 0.05 was considered statistically significant. The statistical analysis was performed using MedCalc^®^ Statistical Software version 22.021 (MedCalc Software Ltd., Ostend, Belgium).

## 3. Results

Our cohort included 736 patients with AKI. A total of 125 children developed AKD representing 17%. The demographics and baseline characteristics of the patients are presented in Table 1. Patients who developed AKD were older, and naturally presented a higher weight, height and body mass index compared with non-AKD ones. The baseline serum creatinine (SCr) was higher in the AKD group; however, there were no statistical differences regarding eGFR between the AKD and non-AKD children. Patients with AKD presented higher maximum SCr, urea levels, uric acid and C reactive protein and lower serum proteins, haemoglobin and sodium levels. The distribution on AKI stages was different between the two groups (*p* < 0.0001). Severe AKI (stages 2 and 3) was present in over half the patients from the non-AKD group, while in patients who developed AKD, AKD stages 2 and 3 comprised more than 78%. Similarly, the incidence of AKI stage 1 was higher in the non-AKD group, comprising 41% of the AKI cases as compared to the AKD group where AKD incidence was inversely correlated with AKD severity. These results were translated into higher ICU admissions in patients with AKD when compared to non-AKD (44% versus 30.6%, *p* = 0.003), and longer hospital stay as well (17 days versus 8 days, *p* < 0.0001). In addition, the incidence of renal replacement therapy was higher in patients from the AKD group (12 patients versus four, *p* < 0.0001).

Next, we evaluated the impact of exposures and susceptibilities on AKD development—Table 2. Among the explored exposures, only sepsis, critical illness and nephrotoxic medications increased the risk of AKD by 2, 1.9 and 2.5 times, respectively, in the crude analysis. From susceptibilities, the presence of CKD increased the risk of AKD by 2 times, neoplasia by 2.7 times, anaemia by 2.9 times, heart failure by 4 times, arterial hypertension by 3.6 times, stem cell transplant by 2.7 times and female gender by 1.47 times in the crude analysis.

We evaluated the impact of AKI causes on AKD development. It seemed that only intrinsic causes increased the risk of AKD. Thus, renal microvasculature alterations, glomerulonephritis, acute tubular necrosis and acute tubule-interstitial nephritis generated odds ratio of 6.8, 4.7, 5 and 3.3, respectively, in the crude analysis—Table 3.

In order to verify if the exposures, susceptibilities, AKI cause and several biological parameters are indeed independent factors that increase the risk of AKD development, we performed a logistic regression with AKD as the dependent variable and the following factors as independent ones: sex, age, baseline creatinine, serum proteins, C reactive protein, sodium, ICU admission, sepsis, critical illness, nephrotoxins exposure, neoplasia, anaemia, heart failure, arterial hypertension, stem cell transplantation and AKI cause (as seen in Table 3). The regression model was fair (AUC = 0.697, 95%CI = 0.639–0.751, Nagelkerke R square = 0.14); only anaemia, glomerulonephritis and acute tubule-interstitial nephritis increased the risk of AKD by 2.47 (95%CI = 1.32–4.59, *p* = 0.0042), 4.94 (95%CI = 1.58–15.4, *p* = 0.0059) and 2.76 (95%CI = 1.55–4.9, *p* = 0.0005) times, respectively, while male gender proved to be protective (OR = 0.56, 95%CI = 0.32–0.97, *p* = 0.04)—Supplementary Materials Table S1.

We further performed an analysis on AKD stages—Table 4. It seems that AKD severity follows AKI severity as 79% of patients with AKD stage 3 presented a prior AKI stage 3 episode, and 61.5% of children with AKD stage 2 had AKI stage 2. In AKD stage 1, the distribution was more even, with around 40% of stage 1 and 2 AKD presenting stage 1 and 2 of AKI and only 18% of AKD stage 1 patients derived from AKI stage 3. The ICU admission incidence was higher in patients with AKD stage 3 compared with all the other stages. Interestingly, baseline SCr was higher only in AKD stage 1 compared to AKD stage 2 and eGFR was similar between the groups. In addition, there were no statistical differences regarding AKD duration stratified by AKD stages. Subsequent AKI event development was similar between AKD and non-AKD patients and also between AKD stages.

The overall mortality was 9.5% (70 cases). The distribution was different regarding the timeframe. In the first 7 days of AKI, there were 16 cases. For a better evaluation of AKD impact on mortality, we excluded from the analysis patients who died during the first 7 days because they could not theoretically reach an AKD state. The cohort for mortality analysis consisted of 720 patients. In this cohort, the mortality was 7.5% (54 deaths). We divided the mortality timeframe during hospitalization and after discharge. There were 36 events during hospitalization and 18 after discharge. The crude analysis and the adjusted analysis for age, sex, environment, ICU admission, subsequent AKI and AKI stages are presented in Table 4. AKD increased the overall mortality risk by 3.7 times in the crude analysis and 2.65 times in the adjusted analysis. In all the subgroup analyses, AKD increased the risk of death (except the adjusted mortality after discharge where it did not reach statistical significance)—Table 5.

**Figure 1:**
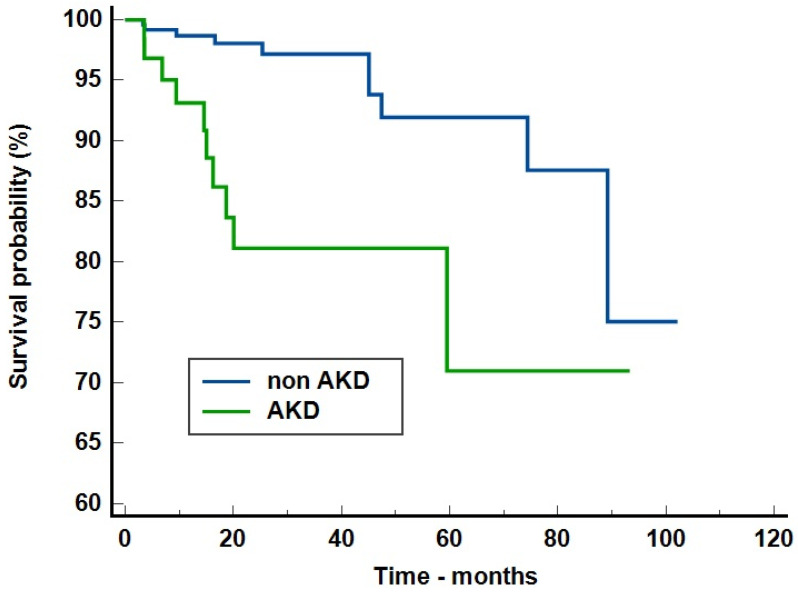
Kaplan–Meier survival analysis on the risk of new-onset chronic kidney disease. Legend Figure 1: AKD = acute kidney disease.

In order to evaluate the risk of new-onset CKD, we included in the analysis only the patients with a follow-up longer than three months and without CKD at admission—313 patients. The median follow-up was at 22.6 months (IQR = 11–41.5 months). The crude analysis of new-onset CKD was performed using the Kaplan–Meier survival curves. The number of censored cases was 10 for non-AKD patients (4.05%) and 10 for AKD group (15.15%). Overall, 20 patients (6.39%) progressed towards new-onset CKD. The presence of AKD increased the risk of new-onset CKD by 7.07 times (95%CI = 2.33–21.44, *p* = 0.0005)—Figure 1.

**Figure 2:**
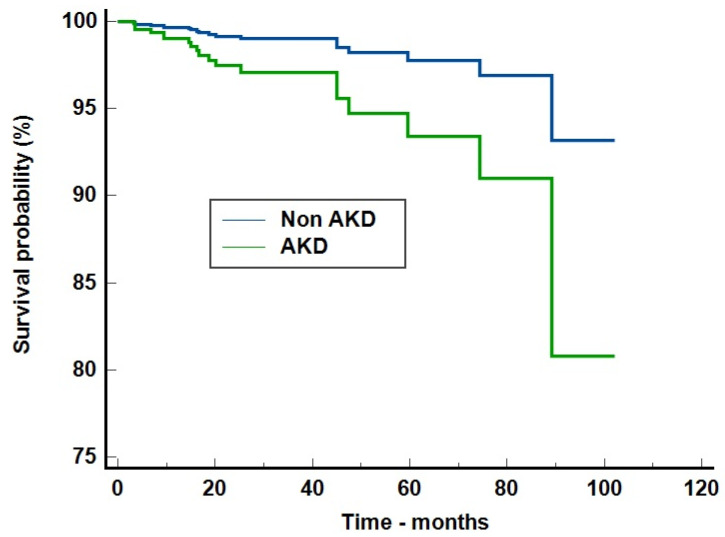
Cox proportional-hazards regression model on the risk of new-onset chronic kidney disease stratified by acute kidney disease presence. Legend Figure 2: AKD = acute kidney disease.

In order to evaluate if AKD was an independent risk factor for new-onset CKD, we performed a Cox proportional-hazards regression analysis. The model was adjusted for age, sex, environment, ICU admission, subsequent AKI, AKI severity, AKI causes and baseline serum creatinine values. The model was good, with a Harrell’s C index of 0.696 (95%CI = 0.522–0.871). AKD remained an independent risk factor for new-onset CKD, increasing the risk by 3.01 times (95%CI = 1.18–7.71. *p* = 0.021)—Figure 2. In this model, only acute tubular necrosis as cause of acute kidney injury increased the risk of CKD (OR = 22.96, 95%CI = 2.38–220.98, *p* = 0.006 with dehydration considered the baseline) and baseline serum creatinine (each unit increase in mg/dL generated an OR = 26.64, 95%CI = 3.42–207.49, *p* = 0.0017).

## 4. Discussions

To the best of our knowledge, this is the first study from Europe that evaluated AKD following an AKI episode in children using the KDIGO criteria and third in the world, after Deng’s study from China [17] and Patel’s study from USA [13]. In this study, we showed that AKD is quite common among children aged from 2 to 18 years old, following an AKI episode, and is associated with worse clinical and biological markers. In addition, the presence of AKD increases both in-hospital and post-discharge mortality. The intrinsic causes of AKI increased the risk of AKD development. AKD was an independent risk for worse outcomes, with high mortality rates (in hospital and post discharge) and an increased risk of progression towards new-onset CKD in children.

The incidence of AKD in our cohort was 17%. This was consistent with previous reported data in children from 1 month to 18 years old where the incidence of AKD ranged between 6 and 56% [13,14,15,16,17]. However, the data are heterogeneous and comprised patients from a wider age spectrum. Yet, Patel [14], LoBasso [15] and Daraskevicius [16] reported AKD incidences of 13%, 11% and 35.3%, respectively, in specific paediatric patients. Patel evaluated patients with non-kidney solid organ transplantation [14], LoBasso children following cardiopulmonary bypass [15] and Daraskevicius children after allogeneic hematopoietic stem cell transplantation [16]. As we mentioned above, there are only two studies that evaluated mixed paediatric patient cohorts, including children between 1 month and 18 years old. One study, by Deng, reported an AKD incidence of 42.3% in 990 AKI children from China [17] and the other study, by Patel, reported a 56.3% incidence [13]. Our reported AKD incidence is lower compared to Patel and Deng. These differences can be explained by the geographic area of the studies (Patel from USA and Deng from China). On the other hand, we included only patients older than 2 years. We chose this approach because it is considered that by the age of 2 years old, children reach full kidney function with an eGFR similar to adults [19].

Regarding baseline characteristics, the patients from our cohort who developed AKD were older. Interestingly, they also presented a higher body mass index when compared with non-AKD ones. Deng is the only one who reported that higher age was more common in patients with AKD [17], while Patel reported similar ages between children with or without AKD [13]. We report higher ages in patients with AKD compared to Deng [17] (6 years AKD stage 1 and 9 years AKD stage 2–3), and Patel (3.8 years) [13]. The only one who reported BMI was Patel, 15.4 in patients with AKD, without statistical differences among groups [13]. A possible explanation for our higher BMI (18.75) would be the older age of the patients. Older children tend to have higher chances of reaching increased BMIs as the incidence of obesity increases with age [23]. The relationship between obesity and kidney disease is not as clear as it is in adults. The most recent review on this topic by Carullo showed that childhood obesity is associated with a higher risk of CKD in adults, but the impact before 18 years old is not clear [24].

The AKD group presented with a more severe underlying disease translated into biological parameters alterations. Thus, patients with AKD had higher uric acid and C reactive protein and lower haemoglobin and sodium. For instance, in Deng’s cohort, there were no differences regarding anaemia incidence, but he found a higher incidence of hypoalbuminemia in AKD patients, especially in AKD stages 2 and 3 (48.9% vs. 23.3% in non AKD children) [17]. The presence of certain exposures and susceptibilities could be a complementary tool in assessing the disease severity in children, besides the known risk factors for AKI development. Patients from the AKD group were found to be at a higher risk for kidney damage when exposed to sepsis, critical illness and nephrotoxic medication and in the presence of certain susceptibilities (neoplasia, anaemia, heart failure, arterial hypertension and stem cell transplant) in the crude analysis. In the adjusted model, only anaemia increased the risk of AKD development. We identified a higher ICU admissions rate in the AKD group (44% vs. 30.6%) and longer hospital stay (17 vs. 8 days). Deng reported similar results in patients with sepsis and exposure to nephrotoxins in both AKD and non-AKD groups, but with a higher incidence of heart failure in patients from the AKD group [17]. Patel reported mechanical ventilation, prematurity, neoplasia and bone marrow transplant as risk factors for AKD development [13].

The kidney disease history and kidney function per se proved to influence the evolution from AKI to AKD. We report a high incidence of CKD among AKD patients (12% vs. 6.5% in non-AKD group) and higher baseline serum creatinine levels. There is a continuum in patients who develop AKI that is dependent on the underlying disease severity. Severe AKI tends to progress to severe AKD, and AKD, regardless of its severity, is an independent risk factor for CKD development. In addition, the intrinsic etiologies of AKI increase the risk of prolonged kidney injury (glomerulonephritis and acute tubule-interstitial nephritis). Yet, our results do not support AKI and AKD severity to be risk factors for progression towards CKD. However, only Deng and Patel [13,17] have reported the relationship between patients with pre-existent CKD and an overlapping AKI or AKD episode. Patients with reduced kidney function are at increased risk of developing a more severe and prolonged AKI episode [6]. So far, CKD is a proven risk factor for AKI and AKD development, at least in adult settings [21].

We report AKD to be an independent mortality risk factor during hospital stay, similar to previous studies [15,17,25]. However, mortality rates in children with AKD are heterogeneous and inconsistent. Deng reported a 12% and 15.9% mortality rate in children with AKD at 30 and 90 days, respectively [17], while LoBasso reported a 31.8% mortality rate after cardio-pulmonary by-pass [15]. Our 17.6% seems to reflect the mortality rates in children with AKD considering that we included patients who reached adult eGFR; however, this is similar to 17.5% mortality rates in children with severe malaria [25].

The final analysis in our study referred to the risk of new-onset CKD. The crude analysis showed that AKD increases the risk of new-onset CKD by seven times and in the adjusted one, by three times. In addition, we showed that acute tubular necrosis increased the risk of new-onset CKD as well as higher baseline creatinine. Similarly, Patel reported the risk of new-onset CKD to be 2.7 times greater in patients with AKD [13]. However, patients with AKD following non-kidney solid organ transplantation had a 29 times higher risk of developing CKD [14]. Interestingly, similar to previous reported data, AKI severity did not increase the risk of CKD [13]. Subsequent AKI episodes did not increase the risk of new-onset CKD, even though Patel found previous AKI episodes to be associated with a higher risk of CKD development [13].

The major limitations are regarding the nature of the study—single-centre, retrospective and observational. On the other hand, the lack of urine output in diagnosing and staging AKI is a drawback. The number of patients, the long follow-up and the individual evaluation of AKD impact on mortality and CKD represent the strong points. In addition, exploring the AKI causes and their relationship to AKD development increases the quality of the paper. Nevertheless, our study is the first one that evaluates AKD incidence and outcomes in a mixed group of paediatric patients from Europe, following an AKI episode, in children older than 2 years.

Children who develop AKD have a higher mortality risk that seems to persist even after discharge. AKD is an independent risk factor for new-onset CKD. Attention should focus on children with pre-existent CKD, as these patients are more susceptible to prolonged and severe kidney injury. Follow-up at least 3 months after an AKI episode should assess kidney function markers and urinalysis.

## Figures and Tables

**Table 1:** Demographics and baseline characteristics of patients with and without AKD.

Variable		AKD N = 125	Non-AKD N = 611	Total N = 736	*p* Value
Gender—male		56 (44.8%)	333 (54.5%)	389 (52.9%)	0.047
Environment—urban		67 (53.6%)	331 (54.2%)	398 (54.1%)	0.906
Age—years M + IQR		12 (8–15)	8 (4–14)	9 (4–14)	<0.0001
Weight—kg (M + IQR)		44 (25–59.25)	26 (16–50)	29 (16–52)	<0.0001
Height—cm (M + IQR)		154 (134.75–164.25)	126 (103–154)	131 (104–158)	<0.0001
BMI—kg/sm (M + IQR)		18.75 (15.48–23.91)	17.04 (14.12–20.78)	17.35 (14.31–21.59)	0.0125
Maximum SCr mg/dL (M + IQR)		1.41 (0.9–3.05)	0.87 (0.61–1.18)	0.92 (0.63–1.34)	<0.0001
Baseline SCr mg/dL (M + IQR)		0.43 (0.31–0.7)	0.36 (0.27–0.54)	0.37 (0.28–0.56)	0.0005
GFR mL/min/1.73 sm (M + IQR)		133.5 (83.2–174.3)	138.4 (109.5–174.2)	137.7 (106.6–174)	0.087
Urea mmol/L (M + IQR)		10.5 (6.4–23.21)	5.75 (4.34–8.2)	6.11 (4.45–9.76)	<0.0001
Uric acid μmol/L (M + IQR)		391 (259.5–618.5)	284 (203.75–400)	297 (217–441)	<0.0001
Serum proteins g/L (M + IQR)		56 (44.55–65.4)	60.2 (51.02–65.87)	59.5 (49.6–65.77)	0.031
Procalcitonin ng/mL		2.13 (0.48–19.12)	3.08 (0.37–17.86)	3.03 (0.4–18.21)	0.866
C reactive protein mg/L (M + IQR)		59.92 (11.41–184.05)	28.73 (3.74–132.4)	35.73 (4.58–14.06)	0.004
Sodium mmol/L (M + IQR)		134 (131–138)	137 (134–139)	137 (133–139)	0.001
Potassium mmol/L (M + IQR)		4.3 (3.6–5)	4.3 (3.8–4.8)	4.3 (3.8–4.8)	0.71
Haemoglobin g/dL (M + IQR)		9.1 (7.6–11.2)	11.05 (9.2–12.5)	10.8 (8.8–12.3)	<0.0001
AKI stage	1	27 (21.6%)	275 (45%)	302 (41%)	<0.0001
	2	44 (35.2%)	195 (31.9%)	239 (32.5%)	
	3	54 (43.2%)	414 (23.1%)	195 (26.5%)	
Hospital stay days (M + IQR)		17 (9.75–31.5)	8 (4–15)	9 (5–18)	<0.0001
IH AKI		67 (53.6%)	353 (57.8%)	420 (57.1%)	0.39
ICU admission		55 (44%)	187 (30.6%)	242 (32.9%)	0.003
RRT necessity		12 (9.6%)	4 (0.7%)	16 (2.2%)	<0.0001

Legend: AKD = acute kidney disease, N = number, M + IQR = median and interquartile range, kg = kilograms, cm = centimeter, sm = square meter, SCr = serum creatinine, mg = milligrams, dL = deciliter, μmol = micromoles, L = liter, g = grams, mmol = millimoles, AKI = acute kidney injury, IH = in hospital, ICU = intensive care unit. Statistical tests: Chi-square test or Mann–Whitney test as appropriate, RRT = renal replacement therapy.

**Table 2:** Exposure and susceptibility distribution in patients with and without AKD.

	Parameter	AKD N = 125	Non-AKD N = 611	Total N = 736	OR and 95%CI	*p* Value
Exposures	Mechanical ventilation	36 (28.8%)	136 (22.3%)	172 (23.4%)	1.41 (0.91–2.17)	0.115
	Sepsis	57 (45.6%)	178 (29.1%)	235 (31.9%)	2.03 (1.37–3.02)	0.0003
	Critical illness	17 (13.6%)	45 (7.4%)	62 (8.4%)	1.97 (1.09–2.65)	0.022
	Hypovolemic shock	3 (2.4%)	18 (2.9%)	21 (2.9%)	0.81 (0.23–2.79)	0.738
	Trauma	2 (1.6%)	35 (5.7%)	37 (5%)	0.26 (0.06–1.12)	0.054
	Major non-cardiac surgery	10 (8%)	62 (10.1%)	72 (9.8%)	0.77 (0.38–1.54)	0.461
	Nephrotoxins	49 (39.2%)	122 (20%)	171 (23.2%)	2.58 (1.71–3.89)	<0.0001
	Poisonous plants	3 (2.4%)	12 (2%)	15 (2%)	1.22 (0.34–4.41)	0.735
Susceptibilities	Dehydration/Volume depletion	115 (92%)	584 (95.6%)	699 (95%)	0.53 (0.25–1.12)	0.095
	Chronic kidney disease	15 (12%)	39 (6.4%)	54 (7.3%)	2 (1.06–2.73)	0.028
	Chronic disease (heart, liver and lung)	19 (15.2%)	68 (11.1%)	87 (11.8%)	1.43 (0.83–2.47)	0.199
	Diabetes mellitus	3 (2.4%)	25 (4.1%)	28 (3.8%)	0.57 (0.17–1.93)	0.368
	Neoplasia	41 (32.8%)	93 (15.2%)	134 (18.2%)	2.71 (1.76–4.19)	<0.0001
	Anaemia	92 (73.6%)	295 (48.3%)	387 (52.6%)	2.98 (1.94–4.58)	<0.0001
	Heart failure	27 (21.6%)	39 (6.4%)	66 (9%)	4.04 (2.36–6.9)	<0.0001
	Arterial hypertension	26 (20.8%)	41 (6.7%)	67 (9.1%)	3.65 (2.13–6.23)	<0.0001
	Stem cell transplant	8 (6.4%)	15 (2.5%)	23 (3.1%)	2.71 (1.12–6.55)	0.021
	Female gender	69 (55.2%)	278 (45.5%)	347 (47.1%)	1.47 (1–2.17)	0.047

Legend: AKD = acute kidney disease, N = number. The statistical test is the Chi-square test.

**Table 3:** AKI causes and AKD development.

AKI Cause		OR (95%CI)	*p* Value
Pre-renal	Hypovolaemia/dehydration	0.11 (0.06–0.19)	<0.0001
	Systemic vasodilatation	1.25 (0.76–2.06)	0.361
	Hypoxia/Ischaemia	0.74 (0.25–2.17)	0.588
Renal	Renal microvasculature alterations	6.89 (2.34–20.23)	0.0004
	Glomerulonephritis	4.74 (1.96–11.42)	0.0005
	Acute tubular necrosis	5.01 (1.23–20.33)	0.023
	Acute tubule-interstitial nephritis	3.39 (2.26–5.08)	<0.0001
Post-renal	Urinary tract malformation/obstruction	0.97 (0.32–2.9)	0.966

Legend: OR = odds ratio, 95%CI = 95% confidence interval.

**Table 4:** Acute kidney disease stages analysis.

		Non AKD N = 611 (83%)	AKD Stage 1 N = 55 (7.5%)	AKD Stage 2 N = 26 (3.5%)	AKD Stage 3 N = 44 (6%)	*p* Value
AKI	AKI stage 1 N = 302 (41%)	275 (45%)	22 (40%)	1 (3.8%)	4 (9.1%)	<0.0001
	AKI stage 2 N = 239 (32.5%)	195 (31.9%)	23 (41.8%)	16 (61.5%)	5 (11.4%)	
	AKI stage 3 N = 195 (26.5%)	141 (23.1%)	10 (18.2%)	9 (34.6%)	35 (79.5%)	
ICU admission		187 (30.6%)	17 (30.9%)	9 (34.6%)	29 (65.9%)	<0.0001
Hospital stay		8 (4–15)	12 (8–24.2)	18 (10–33)	24 (14.5–39.5)	<0.0001
Baseline creatinine		0.36 (0.27–0.54)	0.5 (0.36–0.71)	0.34 (0.27–0.5)	0.39 (0.32–0.77)	0.0005
Baseline GFR		138.4 (109.5–174.2)	125.5 (88.3–173.2)	140.2 (95.3–214.3)	132.7 (69.3–170.9)	0.207
AKD duration		-	6 (3–19.75)	8.5 (3–20)	11 (5–23)	0.354
Subsequent AKI events		54 (8.8%)	3 (5.5%)	3 (11.5%)	3 (6.8%)	0.752

Legend: AKD = acute kidney disease, AKI = acute kidney injury, N = number, statistical analysis: Chi-square test or Kruskal–Wallis test as appropriate.

**Table 5:** Acute kidney disease influence on mortality.

	Crude Analysis OR and 95%CI	Adjusted Analysis OR and 95%CI
Mortality risk during hospitalization	4.22 (2.11–8.4), *p* < 0.0001	2.62 (1.14–5.99), *p* = 0.0221
Mortality risk after discharge	2.73 (1.003–7.44), *p* = 0.049	2.47 (0.901–6.81), *p* = 0.07
Overall mortality	3.75 (2.09–6.72), *p* < 0.0001	2.65 (1.38–5.1), *p* = 0.0033

Legend: OR = odds ratio, 95%CI = 95% confidence interval, adjusted for age, sex, environment, intensive care unit admission, acute kidney injury stages and subsequent acute kidney injury. The adjusted performed analysis was performed using logistic regression.

## Data Availability

The data collected for this study will be available for others, at request directly to the corresponding author. The data that will be available are represented by deidentified participant data. The inform consent form and statistical analysis plan will be available at request. The data will be available upon publication. The data will be available at request at the e–mail address ivan.viviana@umft.ro. The data will be shared after direct request and after approval of the proposal by all the authors.

## Supplementary Materials

The following supporting information can be downloaded at: https://www.mdpi.com/article/10.3390/jcm13113145/s1, Table S1: Logistic regression with AKD the dependant variable.
