# Supplementary material for: Acute Kidney Disease following Acute Kidney Injury in Children—A Retrospective Observational Cohort Study on Risk Factors and Outcomes
Source: J Clin Med. 2024 May 27;13(11):3145. doi: 10.3390/jcm13113145 (PMC11172946; doi:10.3390/jcm13113145)
Supplement: Supplementary file 1 [file jcm-13-03145-s001.zip › jcm-2985446-supplementary.pdf]

Supplemental Table S1. logistic regression with AKD the dependant variable

Variable	Coefficient	Standard error	Wald	P value
Gender - male	-.057724	0.28231	4.1807	0.0409
Anaemia	0.90513	0.31644	8.1817	0.0042
Glomerulonephritis	1.59795	0.57984	7.5946	0.0059
Acute tubule-interstitial nephritis	1.01741	0.29242	12.1058	0.0005
Constant	-1.73136	0.34404	25.3256	<0.0001

Legend: The model was fair, with AUC=0.697 (95%CI=0.639-0.751), Nagelkerke R square=0.1418, p<0.0001. The model excluded the following variables: age, baseline creatinine, serum proteins, C reactive protein, sodium, ICU admission, sepsis, critical illness, nephrotoxins exposure, neoplasia, heart failure, arterial hypertension, stem cell transplantation and among AKI causes – dehydration, systemic vasodilatation, hypoxia/ischemia, renal microvasculature alterations, acute tubular necrosis, postrenal cause.

The model generated the following odds ratios:

Variable	Odds ratio	95%CI
Gender - male	0.5614	0.3228-0.9764
Anaemia	2.4723	1.3296-4.5968
Glomerulonephritis	4.9429	1.5864-15.4012
Acute tubule-interstitial nephritis	2.766	1.5594-4.9064

Legend: 95%CI=95% confidence interval.
